# Percolation of collagen stress in a random network model of the alveolar wall

**DOI:** 10.1038/s41598-021-95911-w

**Published:** 2021-08-17

**Authors:** Dylan T. Casey, Samer Bou Jawde, Jacob Herrmann, Vitor Mori, J. Matthew Mahoney, Béla Suki, Jason H. T. Bates

**Affiliations:** 1 Depatment of Medicine, University of Vermont Larner College of Medicine, 149 Beaumont Ave, Burlington, VT 05405 USA; 2 Complex Systems Center, University of Vermont, Burlington, VT USA; 3 Department of Biomedical Engineering, Boston University, Boston, MA USA; 4 Department of Neurological Science, University of Vermont Larner College of Medicine, Burlington, VT USA; 5 The Jackson Laboratory, Bar Harbor, ME USA

**Keywords:** Physiology, Structural biology, Materials science

## Abstract

Fibrotic diseases are characterized by progressive and often irreversible scarring of connective tissue in various organs, leading to substantial changes in tissue mechanics largely as a result of alterations in collagen structure. This is particularly important in the lung because its bulk modulus is so critical to the volume changes that take place during breathing. Nevertheless, it remains unclear how fibrotic abnormalities in the mechanical properties of pulmonary connective tissue can be linked to the stiffening of its individual collagen fibers. To address this question, we developed a network model of randomly oriented collagen and elastin fibers to represent pulmonary alveolar wall tissue. We show that the stress–strain behavior of this model arises via the interactions of collagen and elastin fiber networks and is critically dependent on the relative fiber stiffnesses of the individual collagen and elastin fibers themselves. We also show that the progression from linear to nonlinear stress–strain behavior of the model is associated with the percolation of stress across the collagen fiber network, but that the location of the percolation threshold is influenced by the waviness of collagen fibers.

## Introduction

The mechanical properties of the alveoli in the lungs derive partly from protein fibers in the extracellular matrix (ECM) composed mainly of the structural proteins collagen and elastin. Elastin fibers are highly extensible and continuously strained, whereas collagen fibers are much stiffer and mostly flaccid, giving a wavy appearance, at low tissue strains^1–3^. As strain increases, the collagen fibers straighten and become progressively recruited to bear an increasing fraction of the elastic load^4^. Furthermore, collagen fibers can form bonds within and between themselves via cross-linking, which alter fiber stiffness and the topology of the network, respectively^5^. Networks of collagen and elastin in alveolar walls are random, web-like, and isotropic^6^. Together, they produce nonlinear stress–strain behavior such that alveolar walls demonstrate an exponential increase in stress with increasing strain^7^.

There is evidence that the collagen and elastin networks in alveolar wall tissue are mechanically coupled in some fashion^8^, but exactly how is unclear. Elucidating this multi-component behavior is crucial for understanding how interstitial lung diseases such as pulmonary fibrosis leads to altered lung function and the clinical symptoms arising therefrom. Previous computational models of lung tissue have represented the alveolar parenchyma as a network of springs, each corresponding to an alveolar wall^9–13^, or as polyhedra representing complete alveoli^14–18^. These models have revealed how the destruction of alveolar walls in emphysema^19^, or the stiffening of walls in pulmonary fibrosis^20^, is linked to pathologic alterations in lung compliance and the histologic patterns typical of these diseases. However, these models do not explicitly represent the microscopic stress-bearing structures, namely the protein fibers, in the walls. There is strong evidence that pulmonary fibrosis is characterized more by aberrations in the arrangement of these structural protein fibers rather than by changes in their amounts^21^. Additionally, the role that fiber arrangement plays in bulk tissue mechanics can vary with tissue strain as a result of percolation of fiber stress across the tissue^10^. Percolation in this context refers to the point during stretch at which a contiguous set of recruited fibers suddenly spans the entire network, which forms a giant component of the largest sub-network of connected recruited fibers.

Here we present a computational model of the alveolar wall comprised of networks of randomly oriented collagen and elastin fibers coupled via a tunable degree of inter-network cross-linking. We use this model to determine how the bulk stress–strain behavior of the alveolar wall arises as an emergent consequence of percolation across the collagen network under the modulating influence of the fiber waviness.

## Results

### Model stress–strain behavior

**Figure 1:**
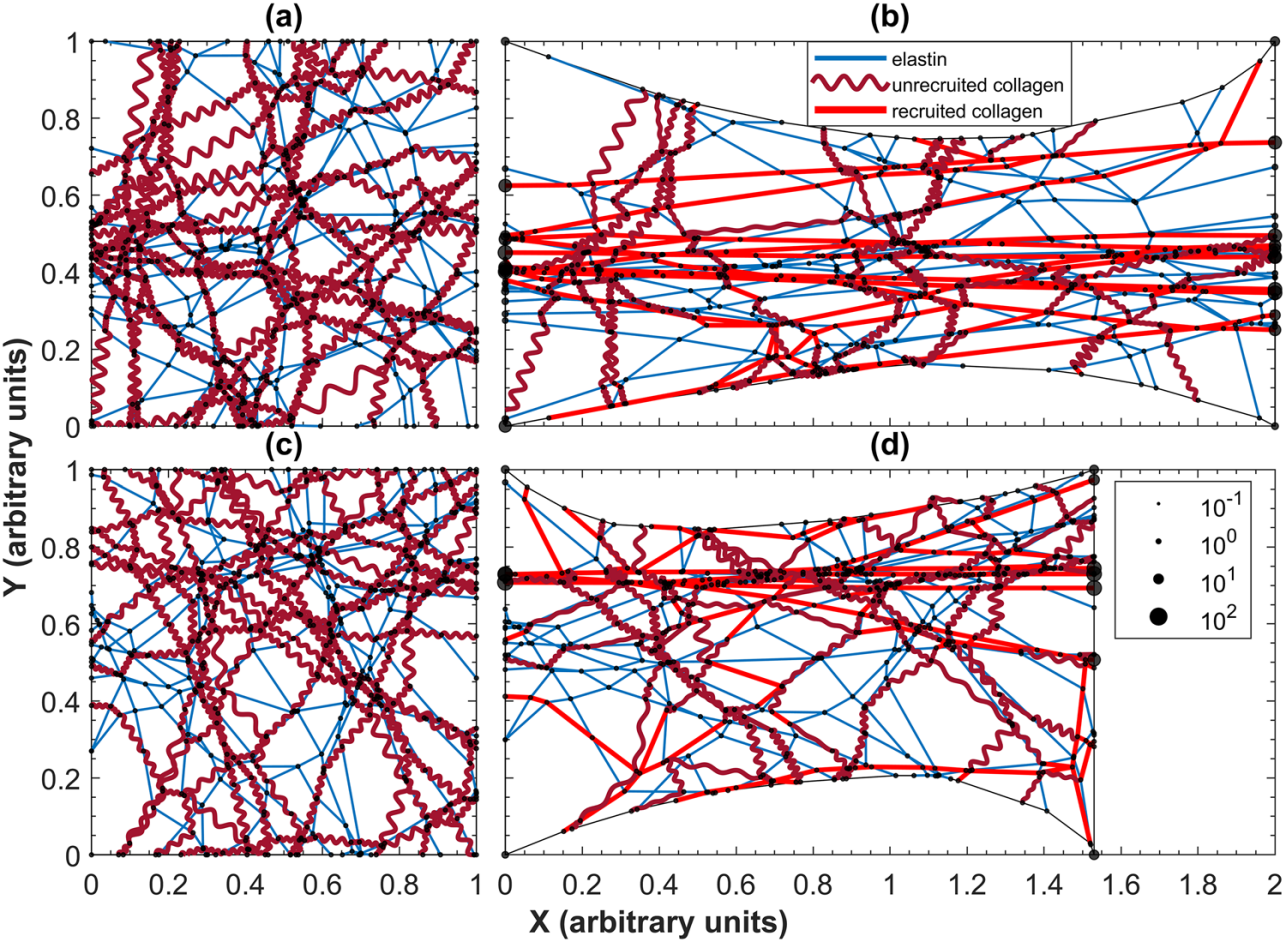
Sample alveolar networks before and after stretching. Elastin fibers are blue and collagen fibers are red. The dark red represents unrecruited collagen, the arc length of each fiber is equivalent to its resting length. Bright red is recruited collagen. Nodes occur where two fibers are cross-linked, and their size represents the net force on the node with logarithmic scaling. The examples shown correspond to: (**a**) initial healthy condition, (**b**) stretched healthy condition, (**c**) initial severe fibrotic condition, and (**d**) stretched fibrotic condition. The maximum stretches in each case reflect those in Fig. 2a. Videos of this stretching process for the healthy and fibrotic conditions can be found in Supplemental Videos 1 and 2, respectively.

**Figure 2:**
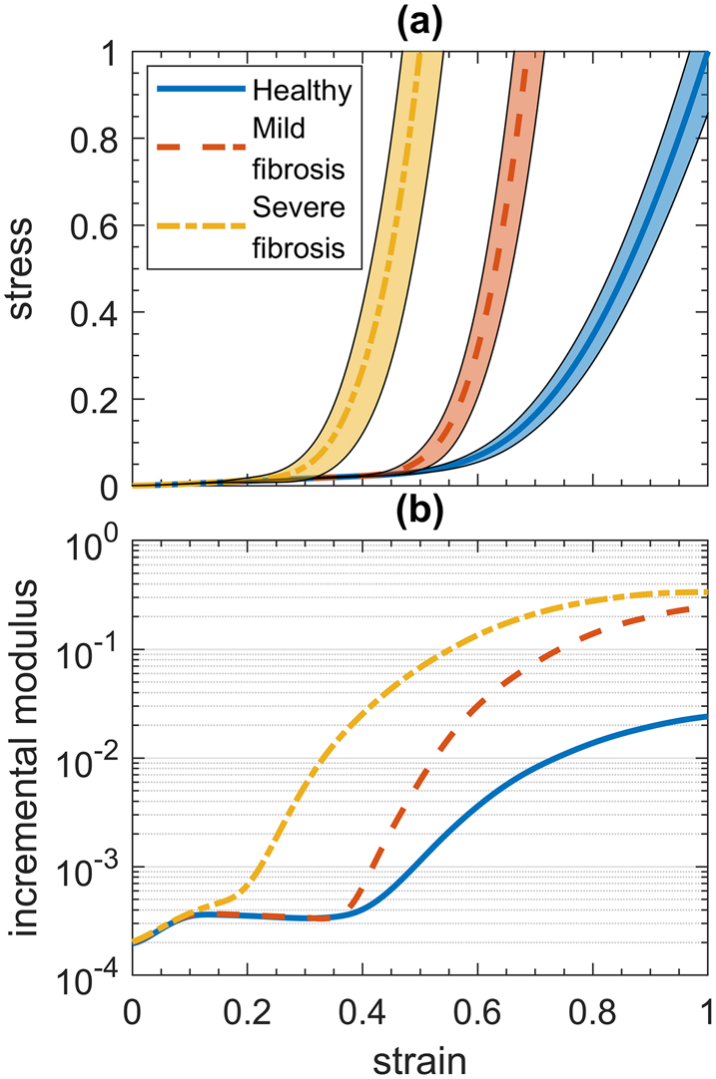
Stress-strain behavior of the model. (**a**) The mean of the stress–strain curves for the three health conditions are marked with shaded regions representing 99% confidence intervals. (**b**) The modulus of the curves in (**a**).

Figure 1 shows examples of healthy and fibrotic networks at baseline and following stretch. The emergent bulk mechanical behavior of our network is illustrated in Fig. 2a, which shows stress–strain relationships corresponding to normal, mildly fibrotic and severely fibrotic alveolar tissue. The model produces highly nonlinear stress–strain curves as a result of the progressive recruitment of initially wavy collagen fibers. The waviness of the fibers is the difference between the arc length of a fiber and its end-to-end distance in situ. Increasing the mean stiffness of the fibers and reducing their waviness both steepens the stress–strain relationship at lower stresses and moves its knee to lower strains. These effects are enhanced by the heavy-tailed Burr distribution of spring constants (Eq. 3), which results in wavier collagen fibers being stiffer than less wavy fibers. The Burr distribution is conveniently customized via its two adjustable parameters so that our model describes the data well. Figure 2b shows the incremental modulus for each condition, each of which journeys toward a plateau. The severe condition makes an early departure from the other conditions at strain 0.1 that head slightly downward until 0.35. The modulus for the severe condition transitions to concave downward at 0.2, while the others transition at 0.35.

### Fiber recruitment and percolation

Early in the stretching process many of the nodes in the model move somewhat chaotically between consecutive stretch states (Supplemental videos 1, 2). Such nodes were attached to only unrecruited collagen fiber segments and so did not have unique minimum energy positions. When several collagen fibers percolated in proximity, they collapsed in on one another, forcefully pulling adjacent fibers inward in accordance with histological images^22^. The healthy condition had more percolated fibers in parallel that shared to total force across the tissue, while in the fibrotic condition there were fewer percolated fibers.

**Figure 3:**
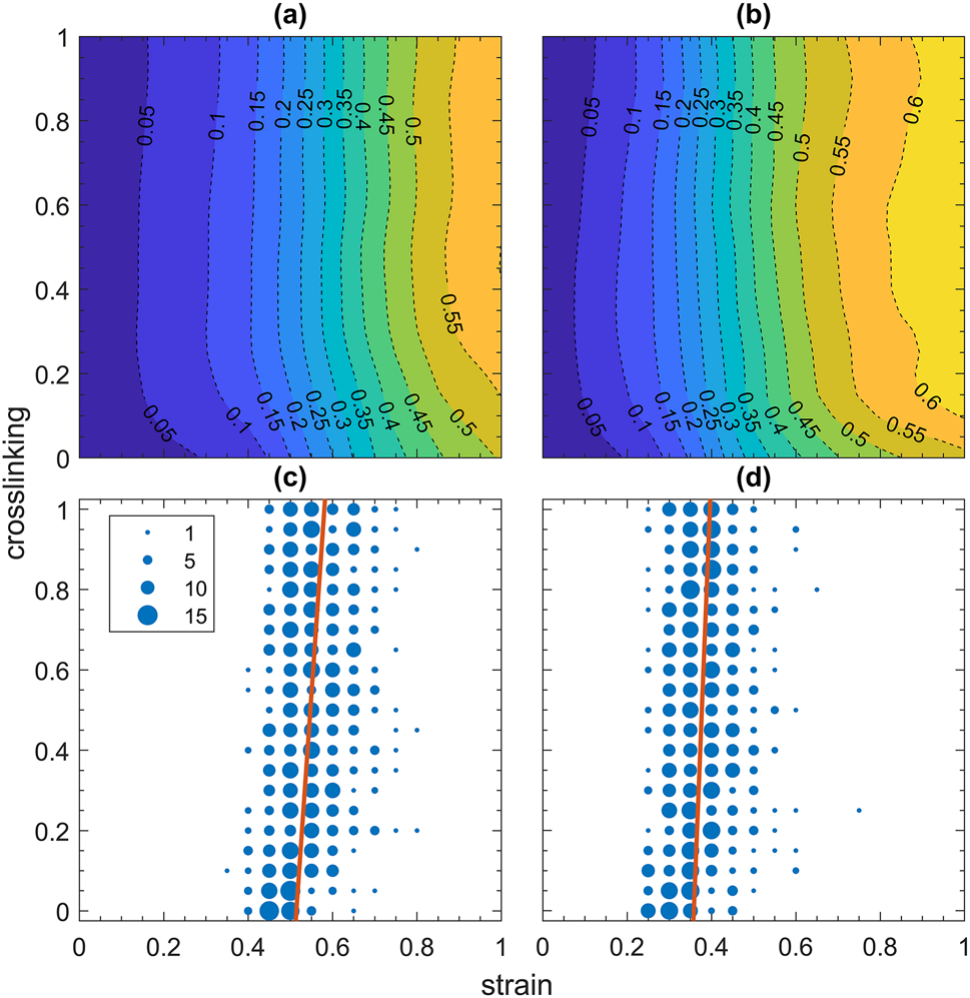
Fraction of collagen recruitment (indicated by the colors and isopleths) as a function of cross-linking ($\rho$) and strain. (**a**) Corresponds to the healthy condition and (**b**) to the severely fibrotic condition. Corresponding plots of the collagen mechanical percolation threshold for (**c**) the healthy condition and (**d**) the severely fibrotic condition. The diameters of the blue circles correspond to the number of simulations exhibiting mechanical percolation at the corresponding values of cross-linking and strain. The orange lines in (**c**,**d**) are trend lines calculated by weighted least squares.

The increased values of $k$ in the fibrotic conditions caused elastic energy to be stored in the system at increased rates as strain developed. Collagen recruitment was not affected by inter-fiber cross-linking above about 30% in the healthy condition and above about 20% in the fibrotic condition (Fig. 3a,b, respectively). The recruitment fraction was slightly greater at a given strain in the fibrotic condition compared to the healthy condition (Fig. 3b versus a).

**Figure 4:**
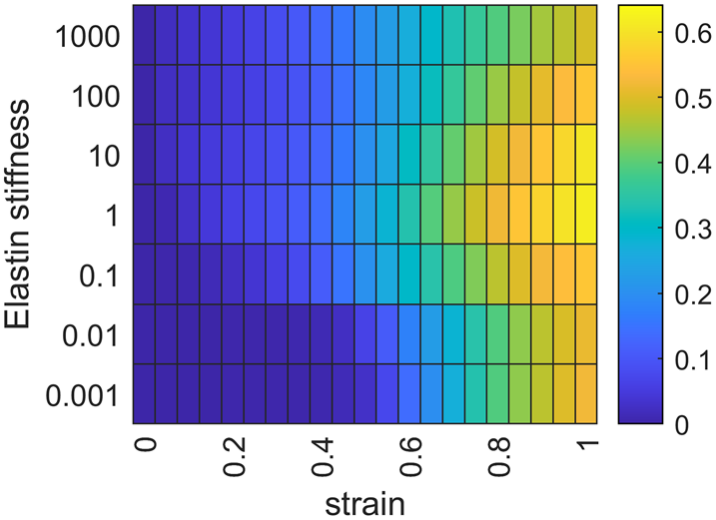
Collagen recruitment fraction (indicated by color according to the color bar at the right) as a function of variation in elastin stiffness relative to its baseline value of 1 (vertical axis) and strain (horizontal axis). At 1000, elastin is as stiff as the stiffest collagen fibers. These results represent the average of 40 independent runs of the model at each value of elastin stiffness.

Interestingly, the baseline value of elastin stiffness (i.e., 1000-fold lower than collagen stiffness^23^) resulted in the earliest peak in collagen recruitment with increasing strain (Fig. 4). This peak occurred at progressively higher degrees of stretch as elastin stiffness either decreased or increased relative to baseline.

**Figure 5:**
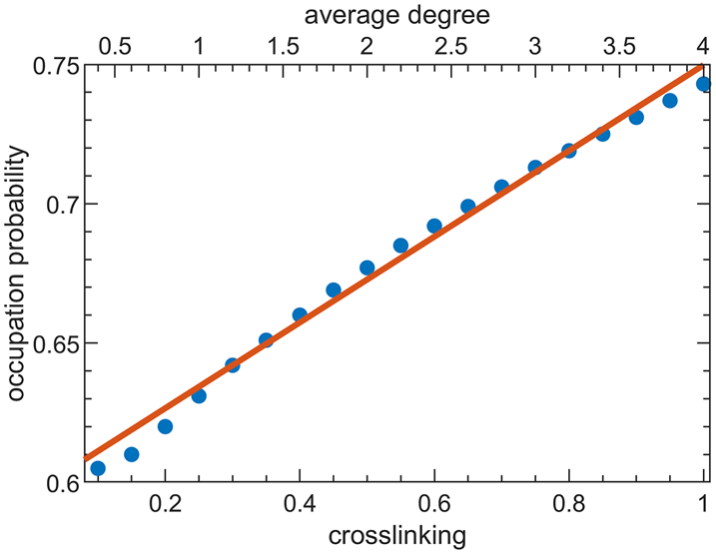
Occupation probability at the bond percolation threshold as a function of cross-linking fraction, $\rho$ (translated into average network degree along the top horizontal axis). The orange line shows the linear fit to these data. The bond percolation thresholds corresponding to the symbols were taken from the inflection points of the curves shown in Fig. 6 respective to their cross-linking values.

**Figure 6:**
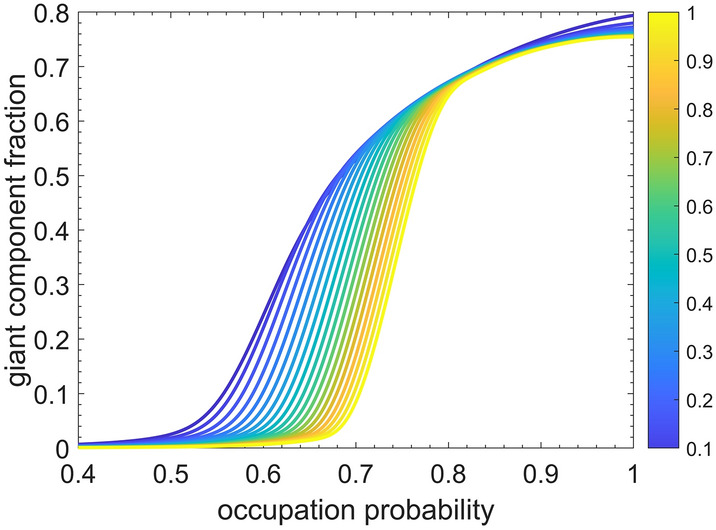
Giant component size with random link occupation for networks of 480 fibers with various cross-linking values as indicated by the line color according to the color bar.

Bond percolation threshold in our model was approximately 0.725 and was independent of network size (Fig. 5). The giant component (the number of segments in the largest connected set) grew as the bond occupation probability on the collagen subnetwork increased, but it never consumed the entire network, even at 100% collagen recruitment, due to the appearance of isolated islands of recruited collagen fibers that constituted larger fiber fractions in networks with more cross-linking (Fig. 6). For every level of cross-linking, bond percolation occurred at substantially higher fractions of collagen fiber occupation (0.60–0.75) compared to the 0.20–0.30 collagen recruitment fraction corresponding to mechanical percolation (Fig. 3c,d).

The bond percolation threshold was close to linearly dependent on the degree of inter-fiber cross-linking (Fig. 5). The relationship broke down below 10% recruitment, however, because of the decreased likelihood of fiber segments being part of the giant component; at 0%, for example, the giant component may comprise only a single fiber.

## Discussion

We have developed a novel random fiber network model of the alveolar wall to investigate the changes taking place in lung tissue mechanics with the development of pulmonary fibrosis. We were able to recreate realistic nonlinear stress–strain curves using only Hookean springs to represent both collagen and elastin (Fig. 2a). Collagen was flaccid and wavy at baseline strain, and gradually became recruited to bear the load as strain increased, with elastin assisting in the recruitment. Increasing collagen spring constants to mimic increased cross-linking^24^ facilitated the transition from healthy to mild fibrosis. Subsequent changes in collagen waviness distribution proved to be essential for the transition from mild to severe fibrosis. Network topology did not change the collagen recruitment fraction (provided the average connectivity degree of the network was at least 1), and nor did the mechanical percolation threshold. We also found that the bond percolation threshold increased with average network degree.

By randomizing the spring constants and resting lengths of the various fibers in the model, we were able to give an irregular yet globally isotropic appearance reminiscent of micrographs of real lung parenchymal sections^6^. The fiber networks generated by our algorithm are always regular degree 4 graphs, and so do not account for branching that can give rise to degree 3 nodes. However, the combined network structure of collagen with elastin in lung tissue is not understood in detail, so we decided to bond fibers at their intersections with a certain probability to simulate inter-fiber cross-linking, and to make these bonds permanent. It is possible, however, when fibers come into contact, the resulting frictional forces might create some form of more transient connection. Such a mechanism of temporary bond formation and subsequent breakage could be a mechanism for viscoelastic behavior, as shown by Bates and Ma^25^.

The necking in of the network is reminiscent of cellular induced compression of the extracellular matrix which may have implications for mechanosensing^26^. Elastin fibers began elongating immediately upon tissue stretch because they start at their resting lengths so the only mechanical percolation phenomenon in our model involves collagen recruitment. However, collagen recruitment is not independent of elastin because force transmitted between two collagen fibers by an interconnecting elastin fiber can recruit the collagen fibers. Interestingly, this means that percolation in our network is possible below an average connectivity degree of 1, unlike other random graphs such as the Erdős–Rényi graph.

None of the bond percolation networks ever became completely connected, even at total occupation and thus never achieved a giant component size fraction of 1. In fact, the size of the giant component decreased as network size increased due to isolated islands of collagen that arose from the randomness of the network structure. Although the bond percolation threshold increased with the average network degree/cross-linking, the mechanical percolation threshold was virtually independent of the cross-link density (indicated by the trend lines in Fig. 3c,d).

Overall, mechanical percolation of collagen occurred when approximately 20–25% of the collagen was recruited (Fig. 3), which was well below the bond percolation thresholds that ranged between 60 and 75% collagen recruitment (Fig. 5). The reason for this is that bond percolation is a random process, whereas mechanical percolation is driven by preferential load transfer between neighboring links resulting in recruitment that minimizes the total energy. Percolation occurred in the fibrotic and healthy condition at strains 5% and 10%, respectively, beyond the maximum value for the minimum waviness (Fig. 3c,d). One might expect mechanical percolation in a network of only collagen fibers to occur when strain reaches the resting length of the shortest collagen fiber spanning the direction of stretch. However, this was not observed in the healthy condition, suggesting that collagen recruitment and percolation is facilitated by interactions between the elastin and collagen networks.

**Figure 7:**
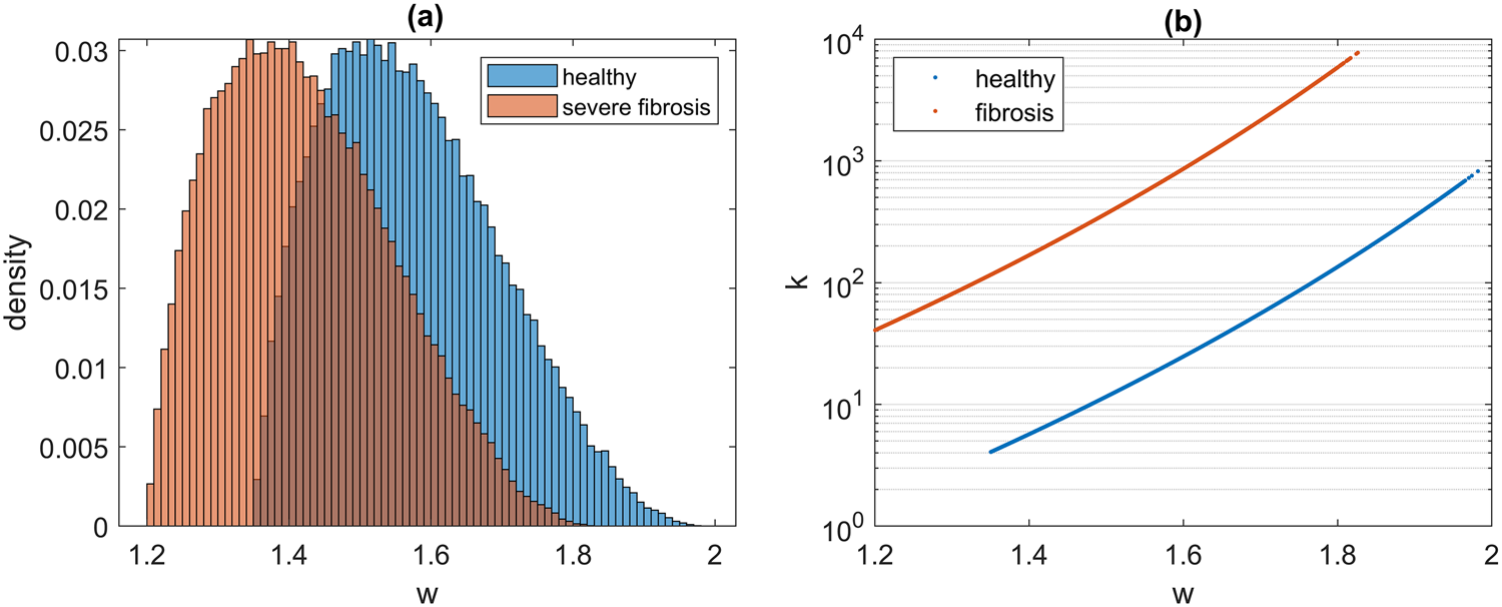
Giant component size with random link occupation for networks of 480 fibers with various cross-linking values as indicated by the line color according to the color bar.

There are very limited data in the literature on the stress–strain curve of the alveolar wall of human subjects, either healthy or diseased. We found only two representative curves that our model was able to recapitulate in overall terms, including changes between healthy and aged tissue^7^. Having a wide variation in collagen waviness is associated with gradual slope variations in stress–strain curves, and decreasing the collagen waviness increases collagen recruitment^27,28^. In models of collagen fiber crimp formation, stiffer fibers are wavier because shear forces buckle the fibers more severely when they are stiffer^29^. We included this mechanism by having collagen spring constants increase with fiber resting length, which improved the fit of the simulated to the experimental data, although at strains well beyond the physiologically relevant range. Via the Burr distribution, spring constants increased with waviness over three orders of magnitude such that wavier fibers were less common, but much more stiff (Fig. 7b). The moduli for the healthy and mild conditions went slightly downward at strains of 0.1–0.35 (Fig. 2b), during which collagen recruitment continued to increase (Fig. 3a,b) suggesting a gradual alignment of collagen fiber segments prior to the percolation threshold. We converted healthy to mildly fibrotic tissue by increasing the collagen spring constants by an order of magnitude, which resulted in maximum strains similar to those found experimentally in young patients with fibrosis^7^. Severe fibrosis, in contrast, was modeled by reducing the minimum value of the collagen waviness but increasing its range, so that ${w}_{1}$ was uniformly distributed between 1.1 and 1.3, in order to capture the heterogeneity of fibrotic disease between patients. The values for minimum collagen waviness for each condition were chosen based on the location of the knee in their respective experimental stress–strain curves^7^.

The fraction of inter-fiber cross-linking, $\rho$, remained at a constant level of 85% in all simulations presented here for consistency. However, we found that increasing $\rho$ to mimic the fibrotic condition had little effect. The randomness of the network made this geometric constraint during stretch insignificant. Intra-fiber cross-linking modeled by an increase in spring-constant values, on the other hand, did affect bulk tissue properties substantially, in line with experimental observations^21^. In addition, the stiffness ratio of elastin to collagen had an optimal value at about 10^–3^ also consistent with observation^23^. Below this value, elastin was not stiff enough to effectively recruit collagen, whereas above this value elastin relieved collagen as the primary bearer of stress.

An obvious limitation of our model is the representation of protein fiber mechanics by Hookean springs, which were chosen for their simplicity. These springs still gave rise to bulk stress–strain behavior that was nonlinear because of the progressive recruitment of collagen over the physiologically relevant strain range, but it is possible that fibers are more accurately represented by strain-stiffening springs. We did not include bending stiffness due to its effects being negligible in soft biological tissues^30^, likely because the length to width ratio of collagen fibers at the scale of an alveolus is so large. Permanent bonds between fibers at their crossing points are also somewhat unrealistic and may have led to artificially high stresses because the bonds were not allowed to break or slip. Isotropy was not varied; however, non-random topologies may affect percolation and other network properties. Various fiber densities were also not investigated, but when coupled with cross-link density may influence network strength^31^. Additionally, real alveolar wall tissue has a finite thickness that was not represented in our model; while modeling the alveolar wall in two dimensions captures the essential features of its geometry, stretch-induced changes in the thickness of alveolar walls could have implications for the function of cells and vessels contained within them.

In conclusion, we have developed a model that represents the interplay of two distinct networks of randomly oriented fibrous proteins having very disparate mechanical properties. The model shows how the incremental modulus of a single alveolar wall reflects the emergent global behavior of these two interacting networks, and that this behavior is driven by the progressive recruitment of stress-bearing collagen fibers with increasing strain. The nonlinear nature of this global behavior is associated with a mechanical percolation threshold while being nearly independent of cross-linking. We also report the novel finding that the tissue strain at which the percolation threshold appears in the collagen network is influenced by the collagen waviness distribution. Finally, the model shows that the stiffening and straightening of individual collagen fibers can lead to an increase in the bulk mechanical stiffness of lung tissue consistent with the phenotype of pulmonary fibrosis.

## Methods

### Random fiber network generation

To produce a random network of line elements, each representing a protein fiber, we began by seeding $n$ points at random locations on the unit square. A single line segment is drawn through each point at an angle selected randomly with equal probability from [0, 2$\pi$). Each line segment is continued in both directions until it intersects two either opposite or adjacent sides of the unit square. Each line segment is assigned to be collagen with probability $\alpha$ or elastin with probability $\left(1-\alpha \right)$. All line segments are attached to the edge of the unit square whenever they cross its boundary, with segments between resulting and corner nodes along an edge being designated elastin. At every intersection within the unit square where two fibers cross, we let the fibers be joined by an inelastic bond with probability $0<\rho <1$ to represent *inter-fiber cross-linking*. Such cross-linking does not affect the identity (collagen versus elastin) of the original fiber on either side of any network node created by cross-linking.

Each fiber segment in the network acts like a Hookean spring having potential energy, $U$, given by

1 $$U=\frac{1}{2}k{\left(d-{r}_{0}\right)}^{2}$$

where $k$ is a spring constant, $d$ is the length of a spring, and ${r}_{0}$ is its resting length. If $d\le {r}_{0}$ for a collagen fiber then it is unrecruited and $U=0$; otherwise, the collagen is recruited. *Intra-fiber cross-linking* of collagen, which causes fibers to become stiffer, is represented by increasing the value of $k$.

To create a baseline model, we let $n=48$ and $\rho =0.85$. This produced an average connectivity degree for the network of 3.4 (note that if $\rho =1$, all fiber–fiber crossings correspond to nodes, so the connectivity degree would be 4)^32^. The network was imbued with an initial degree of isotropy by randomly scaling ${r}_{0}$ for each fiber segment (i.e., each spring connecting between two adjacent nodes) by a factor drawn from a uniform distribution on [0, 1], while setting $k$ to be the inverse of ${r}_{0}$ such that the stiffness of every fiber was unity. The MATLAB 2019b (The Mathworks Inc., Natick, MA, USA) nonlinear solver *fmincon* was used to simultaneously minimize both $U$ (Eq. 1) for each spring and the net force on each node (threshold 10^–2^) via iterative adjustment of the node positions. This generated an initial equilibrium configuration for the spring network.

We then re-defined values for ${r}_{0}$ for each spring based on their respective lengths in the equilibrium configuration as follows. Those springs corresponding to elastin were assigned ${r}_{0}$ values equal to their lengths in the equilibrium configuration. Edge springs were given ${r}_{0}$ values equal to 95% of their lengths in the equilibrium, which caused all edge fibers to be under tension. We made the remaining springs (corresponding to collagen) flaccid by assigning ${r}_{0}$ values given by their lengths in the equilibrium configuration each multiplied by a waviness factor ($w$) randomly chosen from a beta probability distribution function ($p$) as measured by Bou Jawde et al. according to^33^

2 $$p\left(w\right)= \frac{{\left(w-{w}_{1}\right)}^{a-1}{\left({w}_{2}-w\right)}^{b-1}}{\beta \left(a,b\right){\left({w}_{2}-{w}_{1}\right)}^{a+b-1}}$$

where $a=1.89$, $b=3.6$, $\beta \left(a,b\right)$ is a factor that normalizes the total area under $p(w)$ to unity, and the limits of $w$ satisfy ${w}_{2}-{w}_{1}=0.65$ (Fig. 7a). The width of the distribution was kept fixed, but the distribution could be shifted to higher or lower ranges of waviness by adjusting ${w}_{1}$ and ${w}_{2}$. The spring constant, $k$, for each fiber segment was re-initialized as the inverse of its ${r}_{0}$ value. The spring constant of each collagen fiber was then further scaled to account for its waviness factor. A flipped Burr distribution function ($f$) was used according to

3 $$f\left(w|c,\kappa , \gamma \right)= \gamma c\kappa \frac{{\left({w}_{2}-w\right)}^{c-1}}{{\left(1+{\left({w}_{2}-w\right)}^{c}\right)}^{\kappa +1}}$$

where $c=1$ and $\kappa =10$ (Fig. 7b). Finally, the collagen spring constants were amplified by three orders of magnitude to represent healthy tissue ($\gamma ={10}^{3})$ and by four orders of magnitude to represent fibrotic conditions ($\gamma ={10}^{4})$^23^.

### Uniaxial stretch

The network was stretched uniaxially in the horizontal direction, using incremental steps in the position of its right-hand boundary, up to 100% strain in 5% increments. The elastic equilibrium configuration of the network was calculated at each step. The nodes and springs comprising the right and left edges of the network were constrained to remain co-linear during stretching, while the top and bottom edges could deform inwards, as illustrated in Fig. 1. The sum of the nodal forces (arbitrary units) along the right-hand boundary in the direction of the stretch was determined at each step as a measure of the total stretching force applied to the tissue. Stress was calculated as this force divided by the total possible area at its respective step.

### Collagen fiber percolation

Collagen fiber percolation occurs when there is a continuous pathway of recruited collagen fiber segments across the network from the left-hand to the right-hand boundaries. We investigated how this condition arises in two different ways: (1) mechanical percolation, and (2) bond percolation. The mechanical percolation threshold is defined as the strain at which a contiguous pathway of recruited collagen first spans the tissue during network stretching. In contrast, bond percolation occurs simply through the random progressive occupation of links in the collagen network. We investigated bond percolation by generating 4000 networks with $n=480$, and $\rho =0.85$. In each network, we randomly designated segments as occupied with probabilities between 0 and 1 in steps of ${10}^{-3}$.

At each step, we determined the size of the giant component as a fraction of the entire network size. The results from all the networks were averaged at each probability step, and the inflection point of the relationship between probability and the size of the giant component was taken as the bond percolation threshold.

### Modeling healthy versus fibrotic lungs

In order to simulate healthy and mildly fibrotic alveolar tissue, we varied the minimum collagen waviness uniformly between 1.3 and 1.4, whereas in severely fibrotic tissue, it varied between 1.1 and 1.3. The parameters $\alpha$, $n$, and $\rho$ remained the same for all conditions. We performed 3000 simulations of each condition and normalized total force to the mean of the healthy condition at a strain of 1.

The mechanical effects of inter-fiber cross-linking were investigated by varying $\rho$ between 0 and 1 in a set of 40 independent networks for each condition. Recruitment fraction and collagen percolation were determined at each stretch step.

We examined the effects of elastin stiffness on network behavior for a set of 40 independent healthy networks by varying the elastin spring constants from 10^–3^ to 10^3^. At each stretch step, we determined the fraction of recruited collagen fiber segments and assessed whether mechanical percolation had occurred.

## Supplementary Information

Supplementary Video 1.

Supplementary Video 2.
